# Statistical Differences in Set Analysis in Badminton at the RIO 2016 Olympic Games

**DOI:** 10.3389/fpsyg.2019.00731

**Published:** 2019-04-03

**Authors:** Gema Torres-Luque, Ángel Iván Fernández-García, Juan Carlos Blanca-Torres, Miran Kondric, David Cabello-Manrique

**Affiliations:** ^1^Department of Didactics of Musical, Plastic and Body Expression, Universidad de Jaén, Jaén, Spain; ^2^Department of Physiatry and Nursing, Universidad de Zaragoza, Zaragoza, Spain; ^3^Department of Racket Sports, Faculty of Sport, University of Ljubljana, Ljubljana, Slovenia; ^4^Department of Physical Education and Sports, University of Granada, Granada, Spain

**Keywords:** notational analysis, match analysis, racket sports, performance indicators, performance analysis, badminton

## Abstract

The aim of the present study was to determine statistical differences in a set of badminton competition matches in five different modalities with regard to competition level (Group Phase vs. Eliminatory Phase). Data from 453 sets (125 in men’s singles; 108 sets in women’s singles; 77 sets in men’s doubles; 73 in women’s doubles and 70 in mixed doubles) from the RIO 2016 Olympics Games were recorded and classified in two groups of variables to analyze variables related to match (5) and set (15). A descriptive analysis and univariate test (Mann–Whitney U) for non-parametric data were conducted. The results show in men’s and women’s singles all the variables related to match were higher in the Elimination Phase than in the Group Phase (*p* < 0.01). In Sets 1 and 3, the longest set duration, rally and average rally were found in the Elimination Phase than Group Stage (*p* < 0.05). In women’s singles, these differences were also recorded in Set 2. For doubles, the results are more stable among groups. Men’s doubles had a longer duration of the match and set (sets 1 and set 2) (*p* < 0.01), and also scored highest for average rally strokes (sets 1 3) (*p* < 0.05) and shuttles used in the Elimination Phase vs. the Group Phase along the match (*p* < 0.01). In women’s doubles, more shuttles were used in a match in the Elimination than in the Group Phase. Moreover, the same results are established for Set 2, including for average rally. Mixed doubles saw no match going to three sets. However, the greatest differences showed a longer rally and average rally being registered in the Elimination than in the Group Phase. In conclusion, the timing factors of the badminton singles and doubles games were different in the Elimination and Group Phases. This information may help players and coaches prepare and administer different types of workouts or, more specifically, competition schedules adapted to the characteristics of modern badminton.

## Introduction

Badminton has been an Olympic sport since the Barcelona Olympics Games of 1992. It includes five different modalities: men’s singles, women’s singles, men’s doubles, women’s doubles and mixed doubles (Gawin et al., 2015). In addition, in 2006 the rules were changed from playing 3 sets of 15 points to 3 sets of 21 points, with obvious differences being later revealed (Chen and Chen, 2011) and triggering a rise in scientific research in this sport due to the modified technical/tactical, physical and physiological conditions (Phomsoupha and Laffaye, 2015). The main effect in the change of regulation has been a shortening of the times of game as far as the temporary structure (Phomsoupha and Laffaye, 2015). Accordingly, player characteristics in terms of somatotype are currently defined (Abián et al., 2012; Hussain, 2013) to include their physiological characteristics (Alcock and Cable, 2009; Jeyaraman et al., 2012), visual fitness (Williams et al., 2011; Di et al., 2012) or biomechanical aspects (Hussain et al., 2011; Li et al., 2017).

One area capturing most attention from researchers is notational analysis, fundamental in high-level competition such as world-class competition (Gawin et al., 2015) or badminton championships (Abdullahi and Coetzee, 2017). However, most investigations have involved the Olympics Games (Laffaye et al., 2015; Abián-Vicén et al., 2018; Chiminazzo et al., 2018). They have focused on aspects related to the temporal structure, where efforts have considered individual badminton aspects, inter alia establishing: a match duration of 48–65 min (Abian-Vicen et al., 2013; Phomsoupha and Laffaye, 2015; Chiminazzo et al., 2018), point duration of around 9 s (Abian-Vicen et al., 2013; Abián et al., 2014) or number of strokes per point at around 8–10 (Abian-Vicen et al., 2013; Abián et al., 2014). Recently, even differences have been established for game duration and types of stroke between the group phase and eliminatory phase in men’s singles (Chiminazzo et al., 2018).

Yet studies on a modality like doubles are scarcer, noting one recent study that observed an development of time variables over the last three Olympic Games entailing a gradual rise in match duration or playing time, both in men’s doubles (Abián-Vicén et al., 2018). However, there is much less literature concerning women’s doubles and mixed doubles than for the other modalities.

Finally, we note that while the structure of a match has been analyzed (Abián-Vicén et al., 2018; Chiminazzo et al., 2018), only very rarely has the structure of a set (Abian-Vicen et al., 2013; Abián et al., 2014). In the Olympic Games they are classified by World Ranking in five events. The structure of the Olympic Games consists of a group phase, so that later the best of group goes to the elimination phase [Badminton World Federation (BWF), 2017]. To our knowledge, no research has previously considered with respect to the Olympic Games the structure of individual sets or the game phase at such a high level.

The aim of this study was therefore to analyze statistical differences in a set of competition badminton matches in five different modalities with regard to competition level (Group Phase vs. Eliminatory Phase) at the RIO 2016 Olympic Games.

## Materials and Methods

### Sample

Data from 453 sets (125 sets in men’s singles; 108 sets in women’s singles; 77 sets in men’s doubles; 73 in women’s doubles and 70 in mixed doubles) from the RIO 2016 were analyzed. The unit of analysis was the set to prevent differences in comparing Group Phase (GP) (*n* = 344 sets) and Eliminatory Phase (EP) (*n* = 109) matches (Table 1).

**Table 1:** Number of sets analyzed by phase and sex.

	Group phase	Eliminatory phase	Total
Men’s singles	94	31	125
Women’s singles	90	18	108
Men’s doubles	54	23	77
Women’s doubles	52	21	73
Mixed doubles	54	16	70
Total	344	109	453


All matches were played under the current badminton rules, where the person winning the best of 3 games of 21 points is the winner [Badminton World Federation (BWF), 2017]. The sample represents 100% of all matches played. The competition characteristics show the players were the best in the world at the time.

### Procedure

Data were collected from the Olympic Games’ official website^1^ (accessed 10 September 2016) using the same methodology as previous studies (Ortega et al., 2009; Sánchez-Pay et al., 2015; Torres-Luque et al., 2017). The variables analyzed are shown in Table 2.

**Table 2:** Variables studied in doubles badminton competition.

Group of variables	Game statistics
Variables related to match	Duration of match, longest rally (s), longest rally (strokes), average rally (s), average rally (strokes), shuttles used
Variables related to set	Match points, set points, duration of set, total points played, total points won, most consecutive points, longest rally (s), longest rally (strokes), average rally (s), average rally (strokes), shuttles used, points scored without service, points scored with service, biggest lead, biggest comeback to win the game


A specifically designed spreadsheet (Microsoft Excel) was used to collect all the statistics regarding the winning and losing players in the different modalities, which were then exported to the software IBM SPSS version 24.0 (IBM Corp. Armonk, New York, NY, United States). Intra reliability was calculated through observer registering the same values of play (one set) on two occasions separated by a 4 weeks period. Cohen’s Kappa was used and 0.93 was obtained for observer. The value was considered as very good (>0.80) (Landis and Koch, 1977).

### Statistical Analysis

The statistical program for analysis IBM SPSS version 24.0 (IBM Corp. Armonk, New York, NY, United States) was used. First, a descriptive analysis of the data (means and standard deviation) was performed. Second, a univariate (Mann–Whitney U) test (non-parametric) was conducted with the aim to analyze differences between competition level (Group Phase vs. Eliminatory Phase) in each modality because the assumptions of normality and homogeneity of variances were not satisfied. Unfinished matches were not included in the database. Significance was set at *p* < 0.05.

## Results

Tables 3, 4 shows differences between the Group Phase and Eliminatory Phase for both men’s and women’s doubles.

**Table 3:** Descriptive statistics and different between level of competition (Group Stage vs. Elimination Phase) for men singles sets.

	Men singles						
	
	Group stage	Eliminatory phase	*P-*value	*Z*-value	*d*	CV (Group stage)	CV (Elimnation phase)
**Variables related to match**							
Duration of match	43.81 ± 12.10	58.76 ± 18.75	*p* < 0.001	-3.964	1.067	3.62	3.13
Longest rally (s)	43.11 ± 18.04	45.30 ± 10.24	0.10	-1.642	0.133	2.39	4.42
Longest rally (strokes)	39.20 ± 13.02	42.76 ± 9.04	0.02	-2.209	0.293	3.01	4.73
Average rally (s)	9.53 ± 2.58	10.23 ± 1.88	0.61	-0.505	0.288	3.69	5.44
Average rally (strokes)	7.95 ± 1.45	8.92 ± 1.57	0.01	-2.56	0.655	5.48	5.68
Shuttles used	15.18 ± 7.02	22.92 ± 11.38	0.00	-3.221	0.933	2.16	2.01
**Set 1**							
Match points	*–*	*–*	*–*	*–*		*–*	*–*
Set points	1.60 ± 1.00	1.92 ± 1.38	0.37	-0.897	0.29	1.6	1.39
Duration of set	18.13 ± 4.09	21.61 ± 5.37	*p* < 0.001	-2.823	0.784	4.43	4.02
Total points played	33.60 ± 4.50	34.92 ± 4.44	0.26	-1.111	0.294	7.47	7.86
Total point won	16.82 ± 5.27	17.46 ± 4.78	0.57	-0.558	0.124	3.19	3.65
Most consecutive points	3.79 ± 2.18	3.76 ± 2.42	0.73	-0.335	-0.013	1.74	1.55
Longest rally (s)	35.27 ± 13.02	42.30 ± 11.44	*p* < 0.001	-2.732	0.556	2.71	3.7
Longest rally (strokes)	33.11 ± 9.32	37.69 ± 10.44	0.04	-2.002	0.477	3.55	3.61
Average rally (s)	9.72 ± 2.65	10.30 ± 2.34	0.84	-0.196	0.225	3.67	4.4
Average rally (strokes)	8.18 ± 1.53	8.84 ± 2.11	0.16	-1.392	0.391	5.35	4.19
Shuttles used	6.97 ± 3.00	9.30 ± 3.76	*p* < 0.001	-2.95	0.728	2.32	2.47
Points scored without service	8.07 ± 2.44	8.46 ± 3.00	0.77	-0.287	0.151	3.31	2.82
Points scored with service	8.91 ± 4.63	9.00 ± 4.34	0.96	-0.048	0.02	1.92	2.07
Biggest lead	6.46 ± 4.75	5.65 ± 4.31	0.56	-0.581	-0.174	1.36	1.31
Biggest come back to win the game	2.56 ± 1.93	2.40 ± 1.64	0.90	-0.114	-0.086	1.33	1.46
**Set 2**							
Match points	1.80 ± 1.25	1.87 ± 0.64	0.25	-1.142	0.062	1.44	2.92
Set points	1.87 ± 0.83	1.60 ± 0.89	0.52	-0.629	-0.32	2.25	1.8
Duration of set	20.06 ± 4.27	23.84 ± 6.41	0.01	-2.441	0.775	4.7	3.72
Total points played	34.48 ± 4.69	35.53 ± 2.92	0.15	-1.411	0.243	7.35	12.17
Total point won	17.24 ± 5.01	17.76 ± 3.89	0.93	-0.088	0.109	3.44	4.57
Most consecutive points	3.96 ± 2.08	3.07 ± 1.49	0.07	-1.799	-0.456	1.9	2.06
Longest rally (s)	37.11 ± 19.10	37.30 ± 8.18	0.30	-1.021	0.011	1.94	4.56
Longest rally (strokes)	34.04 ± 14.45	36.53 ± 8.24	0.09	-1.683	0.189	2.36	4.43
Average rally (s)	9.90 ± 3.01	10.23 ± 1.60	0.86	-0.168	0.121	3.29	6.39
Average rally (strokes)	8.23 ± 1.76	9.00 ± 1.74	0.24	-1.171	0.439	4.68	5.17
Shuttles used	6.88 ± 3.20	8.53 ± 4.24	0.06	-1.846	0.474	2.15	2.01
Points scored without service	7.96 ± 2.13	9.57 ± 2.24	*p* < 0.001	-3.179	0.746	3.74	4.27
Points scored with service	9.45 ± 4.54	8.19 ± 3.76	0.14	-1.444	-0.289	2.08	2.18
Biggest lead	6.80 ± 4.18	5.70 ± 3.72	0.30	-1.022	-0.27	1.63	1.53
Biggest come back to win the game	3.13 ± 1.65	2.14 ± 0.89	0.15	-1.413	-0.66	1.9	2.4
**Set 3**							
Match points	1.77 ± 0.83	2.20 ± 1.09	0.47	-0.712	0.478	2.13	2.02
Set points	*–*	*–*	*–*	*–*		*–*	*–*
Duration of set	19.75 ± 4.61	29.40 ± 3.80	*p* < 0.001	-3.93	2.18	4.28	7.74
Total points played	31.87 ± 3.36	37.60 ± 4.43	0.30	-1.027	1.57	9.49	849
Total point won	15.93 ± 5.74	18.80 ± 3.39	0.30	-1.027	0.545	2.78	5.55
Most consecutive points	3.80 ± 2.78	3.20 ± 1.03	0.93	-0.085	-0.243	1.37	3.11
Longest rally (s)	33.00 ± 6.83	48.00 ± 7.74	*p* < 0.001	-3.607	2.124	4.83	6.20
Longest rally (strokes)	30.12 ± 8.18	47.00 ± 4.26	*p* < 0.001	-3.803	2.276	3.68	11.03
Average rally (s)	8.75 ± 1.77	12.20 ± 1.81	*p* < 0.001	-3.734	1.938	4.94	6.74
Average rally (strokes)	7.62 ± 1.54	10.80 ± 1.39	*p* < 0.001	-3.84	2.113	4.95	7.77
Shuttles used	7.12 ± 4.27	13.20 ± 6.94	0.01	-2.546	1.203	1.67	1.90
Points scored without service	7.87 ± 2.52	10.30 ± 2.35	0.03	-2.075	0.98	3.12	4.38
Points scored with service	8.60 ± 5.12	8.50 ± 2.92	0.88	-0.139	-0.021	1.68	2.91
Biggest lead	6.78 ± 4.91	4.11 ± 2.47	0.32	-0.985	-0.601	1.38	1.66
Biggest come back to win the game	2.00 ± 1.29	2.75 ± 0.95	0.28	-1.079	0.617	1.55	2.90


**Table 4:** Descriptive statistics and different between level of competition (Group Stage vs. Elimination Phase) for women singles sets.

	Women singles women						
	
	Group stage	Eliminatory phase	*P -*value	*Z-*Value	*d*	CV (Group Stage)	CV (Elimnation Phase)
**Variables related to match**							
Duration of match	40.11 ± 11.88	50.66 ± 13.75	*p* < 0.001	-3.759	0.865	3.38	3.68
Longest rally (s)	36.52 ± 15.05	38.50 ± 7.37	0.07	-1.776	0.14	2.43	5.22
Longest rally (strokes)	28.64 ± 8.35	34.16 ± 9.22	0.01	-2.532	0.65	3.43	3.7
Average rally (s)	9.35 ± 2.61	10.50 ± 1.74	0.02	-2.24	0.462	3.58	6.03
Average rally (strokes)	6.64 ± 1.40	7.58 ± 1.28	p < 0.001	-2.867	0.68	4.74	5.92
Shuttles used	9.59 ± 3.16	14.83 ± 6.22	p < 0.001	-3.978	1.372	3.03	2.38
**Set 1**							
Match points	-	-	*–*	*–*		–	–
Set points	1.59 ± 0.89	1.25 ± 0.62	0.15	-1.437	-0.399	1.79	2.02
Duration of set	17.76 ± 3.92	21.58 ± 4.32	p < 0.001	-3.41	0.958	4.53	5
Total points played	34.47 ± 4.07	35.58 ± 4.20	0.19	-1.306	0.271	8.47	8.47
Total point won	17.23 ± 4.76	17.79 ± 4.43	0.67	-0.414	0.119	3.62	4.02
Most consecutive points	3.54 ± 2.00	3.41 ± 1.93	0.96	-0.049	-0.065	1.77	1.77
Longest rally (s)	31.97 ± 14.43	33.83 ± 6.78	0.06	-1.865	0.138	2.22	4.99
Longest rally (strokes)	25.52 ± 7.26	31.00 ± 9.99	0.02	-2.294	0.706	3.52	3.1
Average rally (s)	9.64 ± 2.90	10.58 ± 1.93	0.16	-1.392	0.34	3.32	5.48
Average rally (strokes)	6.97 ± 1.51	7.83 ± 1.43	p < 0.001	-2.677	0.574	4.62	5.48
Shuttles used	4.66 ± 1.89	6.91 ± 2.48	p < 0.001	-3.868	1.127	2.47	2.79
Points scored without service	8.54 ± 2.09	9.04 ± 2.34	0.49	-0.688	0.235	4.09	3.86
Points scored with service	8.69 ± 4.31	8.75 ± 3.91	0.92	-0.093	0.014	2.02	2.24
Biggest lead	6.17 ± 4.40	5.35 ± 4.09	0.46	-0.73	-0.188	1.4	1.31
Biggest come back to win the game	2.25 ± 1.77	2.28 ± 1.97	0.80	-0.253	0.017	1.27	1.16
**Set 2**							
Match points	1.50 ± 0.73	1.90 ± 1.10	0.31	-1.014	0.499	2.05	1.73
Set points	1.33 ± 0.51	2.50 ± 2.12	0.44	-0.77	1.207	2.61	1.18
Duration of set	17.50 ± 4.33	22.83 ± 3.84	0.00	-5.186	1.253	4.04	5.95
Total points played	33.42 ± 4.12	35.41 ± 3.37	0.02	-2.285	0.496	8.11	10.51
Total point won	16.71 ± 5.20	17.70 ± 4.12	0.57	-0.559	0.196	3.21	4.3
Most consecutive points	3.57 ± 1.98	4.00 ± 2.43	0.54	-0.608	0.209	1.8	1.65
Longest rally (s)	30.57 ± 12.37	34.16 ± 6.98	0.03	-2.145	0.308	2.47	4.89
Longest rally (strokes)	25.26 ± 8.71	28.33 ± 6.57	0.01	-2.34	0.365	2.9	4.31
Average rally (s)	9.52 ± 2.57	10.66 ± 2.01	0.04	-2.028	0.458	3.7	5.30
Average rally (strokes)	6.78 ± 1.52	7.75 ± 1.56	0.01	-2.391	0.635	4.46	4.97
Shuttles used	4.45 ± 1.64	7.33 ± 3.57	p < 0.001	-4.079	1.388	2.71	2.05
Points scored without service	8.19 ± 2.18	8.58 ± 2.20	0.37	-0.895	0.179	3.76	3.9
Points scored with service	8.72 ± 4.80	9.12 ± 3.92	0.78	-0.268	0.086	1.82	2.33
Biggest lead	6.81 ± 4.39	5.75 ± 3.58	0.41	-0.822	-0.248	1.55	1.61
Biggest come back to win the game	2.43 ± 1.34	2.28 ± 1.11	0.91	-0.102	-0.115	1.81	2.05
**Set 3**							
Match points	1.50 ± 0.54	1.50 ± 0.70	1.00	0	0	2.78	2.14
Set points	*–*	*–*	*–*	*–*		*–*	*–*
Duration of set	20.00 ± 3.86	26.50 ± 5.19	0.05	-1.952	1.584	5.18	5.11
Total points played	36.50 ± 2.31	33.50 ± 2.88	0.08	-1.718	-1.011	15.8	11.63
Total point won	18.25 ± 3.30	16.75 ± 5.31	0.65	-0.454	-0.406	5.53	3.15
Most consecutive points	3.58 ± 1.97	3.00 ± 0.81	0.75	-0.311	-0.316	1.82	3.7
Longest rally (s)	30.16 ± 6.39	41.00 ± 6.92	0.01	-2.44	1.673	4.72	5.92
Longest rally (strokes)	26.16 ± 6.13	36.00 ± 5.77	0.02	-2.222	1.62	4.27	6.24
Average rally (s)	10.66 ± 2.93	13.00 ± 2.30	0.21	-1.235	0.824	3.64	5.65
Average rally (strokes)	7.83 ± 1.94	10.50 ± 1.73	0.02	-2.222	1.399	4.04	6.07
Shuttles used	3.33 ± 0.98	3.50 ± 1.73	1.00	0	0.15	3.4	2.02
Points scored without service	9.08 ± 1.44	8.00 ± 1.15	0.20	-1.26	-0.773	6.31	6.96
Points scored with service	9.16 ± 3.18	8.75 ± 5.12	0.85	-0.183	-0.115	2.88	1.71
Biggest lead	4.41 ± 2.46	8.50 ± 3.53	0.13	-1.49	1.537	1.79	2.41
Biggest come back to win the game	2.66 ± 0.81	*–*	*–*	*–*		3.28	*–*


The results show that for men’s and women’s singles all the variables related to match were longer in the Eliminatory than in the Group Phase (*p* < 0.05). In Sets 1 and 3, the longest set duration (*p* < 0.05), rally (*p* < 0.01) and average rally (*p* < 0.05) were recorded in the Eliminatory Phase. In women’s singles, these differences were also found in Set 2 (*p* < 0.05).

Tables 5, 6 presents differences between moments analyzed in the three doubles modalities.

**Table 5:** Descriptive statistics and different between competition level (Group Stage vs. Eliminatory Phase) for men and women doubles sets.

	Men doubles							Women doubles						
		
	Group stage	Eliminatory phase	*P*-Value	*Z*-Value	*d*	CV *(GS)*	CV *(EP)*	Group stage	Eliminatory phase	*P*-value	*Z*-value	*d*	CV *(GS)*	CV *(EP)*
**Variables related to match**														
Duration of match	48.68 ± 17.87	68.94 ± 11.76	*p* < 0.001	-4.03	1.134	2.72	5.86	47.75 ± 13.00	68.62 ± 17.13	*p* < 0.001	-3.695	1.464	3.67	4.01
Longest rally (s)	42.30 ± 19.96	33.94 ± 10.20	0.10	-1.643	-0.473	2.12	3.33	54.33 ± 18.58	51.87 ± 13.77	1.00	-0.000	-0.141	2.92	3.77
Longest rally (strokes)	43.29 ± 10.11	42.41 ± 12.38	0.43	-0.777	-0.081	4.28	3.43	58.41 ± 15.10	56.37 ± 13.98	0.68	-0.404	-0.138	3.87	4.03
Average rally (s)	6.70 ± 2.16	7.23 ± 1.67	0.64	-0.463	0.261	3.1	4.33	10.33 ± 2.23	10.37 ± 1.99	0.87	-0.158	0.018	4.63	5.21
Average rally (strokes)	7.02 ± 1.11	7.76 ± 1.78	0.27	-1.086	0.552	6.32	4.36	9.91 ± 2.06	10.62 ± 1.70	0.18	-1.32	0.361	4.81	6.25
Shuttles used	15.74 ± 6.20	20.82 ± 6.75	0.01	-2.561	0.798	2.54	3.08	11.83 ± 5.06	16.50 ± 7.32	0.04	-2.022	0.81	2.34	2.25
**Set 1**														
Match points	*–*	*–*	*–*	*–*		*–*	*–*	*–*	*–*	*–*	*–*		*–*	*–*
Set points	2.04 ± 1.04	1.77 ± 0.97	0.44	-0.757	-0.265	1.96	1.82	1.56 ± 0.82	2.11 ± 1.96	0.65	-0.443	0.443	1.9	1.08
Duration of set	18.95 ± 4.57	23.00 ± 5.63	0.01	-2.548	0.826	4.15	4.09	20.20 ± 4.13	24.62 ± 6.08	0.01	-2.521	0.932	4.89	4.05
Total points played	35.89 ± 3.51	36.64 ± 2.66	0.37	-0.89	0.228	10.23	13.77	35.37 ± 3.83	37.37 ± 7.20	0.49	-0.686	0.401	9.23	5.19
Total point won	17.87 ± 3.97	18.52 ± 3.28	0.69	-0.39	0.172	4.5	5.65	17.68 ± 4.29	18.68 ± 5.19	0.55	-0.598	0.22	4.12	3.6
Most consecutive points	3.08 ± 1.39	3.17 ± 1.59	0.93	-0.079	0.062	2.22	1.99	3.41 ± 1.69	2.93 ± 1.52	0.25	-1.135	-0.292	2.02	1.93
Longest rally (s)	34.48 ± 18.00	29.58 ± 6.19	0.51	-0.654	-0.316	1.92	4.78	44.83 ± 16.09	43.50 ± 12.92	0.57	-0.559	-0.087	2.79	3.37
Longest rally (strokes)	38.23 ± 9.84	37.00 ± 7.70	0.93	-0.076	-0.133	3.89	4.81	50.45 ± 13.66	46.87 ± 10.09	0.70	-0.373	-0.28	3.69	4.65
Average rally (s)	7.04 ± 2.71	7.70 ± 1.31	0.68	-0.402	0.277	2.6	5.88	10.29 ± 2.74	11.00 ± 2.12	0.65	-0.443	0.275	3.76	5.19
Average rally (strokes)	7.44 ± 1.39	8.58 ± 1.54	0.01	-2.464	0.794	5.35	5.57	10.00 ± 2.25	11.50 ± 1.86	0.02	-2.304	0.698	4.44	6.18
Shuttles used	6.48 ± 2.19	7.58 ± 1.76	0.07	-1.765	0.531	2.96	4.31	5.79 ± 2.49	7.37 ± 2.87	0.03	-2.096	0.608	2.33	2.57
Points scored without service	9.78 ± 1.70	10.64 ± 2.08	0.18	-1.337	0.473	5.75	5.12	9.25 ± 1.75	10.06 ± 3.04	0.53	-0.621	0.371	5.29	3.31
Points scored with service	8.26 ± 3.62	7.88 ± 3.15	0.67	-0.42	-0.109	2.28	2.5	8.43 ± 3.91	8.62 ± 3.84	0.91	-0.109	0.049	2.16	2.24
Biggest lead	5.34 ± 3.43	4.60 ± 3.56	0.41	-0.814	-0.213	1.56	1.29	6.22 ± 3.43	4.86 ± 3.58	0.16	-1.394	-0.392	1.81	1.36
Biggest come back to win the game	2.42 ± 1.78	2.00 ± 1.29	0.61	-0.51	-0.254	1.36	1.55	3.00 ± 1.34	2.71 ± 1.88	0.63	-0.472	-0.192	2.24	1.44
**Set 2**														
Match points	1.50 ± 0.73	1.66 ± 0.57	0.52	-0.635	0.233	2.05	2.91	1.65 ± 0.87	2.50 ± 1.29	0.15	-1.429	0.848	1.9	1.94
Set points	2.75 ± 1.66	1.33 ± 0.51	0.05	-1.906	-0.998	1.66	2.61	2.00 ± 1.00	1.40 ± 0.54	0.30	-1.021	-0.668	2	2.59
Duration of set	20.23 ± 4.94	29.94 ± 11.36	*p* < 0.001	-2.608	1.308	4.1	2.64	21.54 ± 5.10	24.37 ± 3.93	0.06	-1.87	0.589	4.22	6.2
Total points played	36.44 ± 4.08	37.11 ± 2.80	0.17	-1.346	0.179	8.93	13.25	35.91 ± 4.09	36.75 ± 3.49	0.43	-0.781	0.213	8.78	10.53
Total point won	18.12 ± 3.98	18.70 ± 3.19	0.74	-0.321	0.154	4.55	5.86	17.95 ± 4.21	18.37 ± 3.68	0.89	-0.139	0.103	4.26	4.99
Most consecutive points	3.08 ± 1.52	3.17 ± 1.07	0.53	-0.625	0.064	2.03	2.96	3.08 ± 1.74	2.68 ± 1.07	0.68	-0.404	-0.252	1.77	2.5
Longest rally (s)	31.72 ± 7.77	32.33 ± 1.02	0.55	-0.594	0.093	4.08	31.7	44.75 ± 20.17	46.50 ± 12.28	0.47	-0.714	0.095	2.22	3.79
Longest rally (strokes)	35.57 ± 11.34	35.17 ± 15.31	0.54	-0.609	-0.032	3.14	2.3	47.58 ± 18.69	48.37 ± 15.26	0.80	-0.248	0.044	2.55	3.17
Average rally (s)	6.72 ± 2.44	7.78 ± 17.92	0.35	-0.919	0.223	2.75	0.43	10.75 ± 2.72	10.25 ± 1.98	0.30	-1.034	-0.197	3.95	5.18
Average rally (strokes)	7.02 ± 1.37	7.52 ± 2.26	0.74	-0.328	0.298	5.12	3.33	10.12 ± 2.30	10.12 ± 2.21	0.63	-0.475	0	4.4	4.58
Shuttles used	7.17 ± 2.85	7.47 ± 2.06	0.53	-0.614	0.114	2.52	3.63	5.00 ± 2.14	4.87 ± 2.27	1.00	-0.00	-0.06	2.34	2.15
Points scored without service	10.19 ± 2.22	10.23 ± 1.52	0.96	-0.039	0.02	4.59	6.73	10.08 ± 1.96	10.31 ± 1.95	0.62	-0.495	0.118	5.14	5.29
Points scored with service	7.97 ± 3.68	8.47 ± 2.89	0.63	-0.474	0.114	2.17	2.93	8.21 ± 3.55	8.06 ± 3.21	0.86	-0.17	-0.043	2.31	2.51
Biggest lead	4.95 ± 3.29	4.20 ± 2.80	0.55	-0.591	-0.238	1.5	1.5	5.22 ± 3.56	4.06 ± 3.28	0.20	-1.266	-0.333	1.47	1.24
Biggest come back to win the game	2.56 ± 1.15	1.71 ± 1.49	0.05	-1.905	-0.675	2.23	1.15	2.37 ± 1.14	1.57 ± 1.13	0.08	-1.748	-0.703	2.08	1.39
**Set 3**														
Match points	2.28 ± 1.49	1.87 ± 1.12	0.62	-0.492	-0.295	1.53	1.67	2.50 ± 1.91	2.20 ± 1.64	0.89	-0.129	-0.163	1.31	1.34
Set points	*–*	*–*	*–*	*–*		*–*	*–*							
Duration of set	25.00 ± 4.86	22.33 ± 4.65	0.83	-0.207	-0.556	5.14	4.8	23.25 ± 3.15	26.60 ± 3.74	0.07	-1.8	1.008	7.38	7.11
Total points played	38.57 ± 2.70	39.33 ± 5.10	0.46	-0.731	0.213	14.29	7.71	36.25 ± 2.65	36.20 ± 3.91	1.00	-0.00	-0.016	13.68	9.26
Total point won	19.28 ± 2.61	19.66 ± 3.79	0.41	-0.814	0.126	7.39	5.19	18.12 ± 3.60	18.10 ± 4.12	1.00	-0.00	-0.005	5.03	4.39
Most consecutive points	3.00 ± 1.10	3.25 ± 1.21	0.42	-0.806	0.221	2.73	2.69	3.12 ± 1.24	3.22 ± 1.20	0.84	-0.2	0.081	2.52	2.68
Longest rally (s)	34.85 ± 11.62	15.22 ± 3.41	0.00	-3.299	-1.975	3	4.46	46.25 ± 3.05	41.80 ± 10.65	0.28	-1.08	-0.724	15.16	3.92
Longest rally (strokes)	34.85 ± 9.71	29.16 ± 6.78	0.07	-1.76	-0.636	3.59	4.3	52.50 ± 2.44	50.20 ± 14.35	0.47	-0.714	-0.295	21.52	3.5
Average rally (s)	6.07 ± 1.19	6.66 ± 0.98	0.24	-1.166	0.521	5.1	6.8	9.75 ± 1.38	15.50 ± 17.48	0.36	-0.911	0.623	7.07	0.89
Average rally (strokes)	7.71 ± 1.06	6.66 ± 1.15	0.03	-2.118	-0.966	7.27	5.79	10.00 ± 2.26	10.20 ± 1.81	0.85	-0.18	0.093	4.42	5.64
Shuttles used	7.00 ± 1.92	8.16 ± 2.44	0.07	-1.787	0.556	3.65	3.34	6.25 ± 2.76	6.80 ± 3.08	0.58	-0.54	0.193	2.26	2.21
Points scored without service	11.71 ± 1.85	11.08 ± 1.50	0.43	-0.785	-0.359	6.33	7.39	9.75 ± 1.03	9.80 ± 1.47	0.81	-0.231	0.043	9.47	6.67
Points scored with service	7.57 ± 2.65	8.58 ± 2.84	0.28	-1.069	0.373	2.86	3.02	8.37 ± 3.15	9.22 ± 2.38	0.66	-0.437	0.287	2.66	3.87
Biggest lead	4.50 ± 2.75	4.45 ± 2.97	0.85	-0.178	-0.018	1.64	1.5	4.37 ± 3.54	5.71 ± 3.63	0.34	-0.939	0.411	1.23	1.57
Biggest come back to win the game	2.33 ± 2.30	3.40 ± 2.88	0.63	-0.476	0.427	1.01	1.18	1.50 ± 0.57	2.50 ± 0.70	0.13	-1.5	1.644	2.63	3.57


*GS, Group Stage; EP, Elimination Phase.*

**Table 6:** Descriptive statistics and different between competition level (Group Stage vs. Eliminatory Phase) for mixed doubles sets.

	Mixed doubles						
	
	Group stage	Eliminatory phase	*P*-value	*Z*-value	*D*	CV (Group stage)	CV (Elimnation Phase)
**Variables related to match**							
Duration of match	47.45 ± 16.36	44.25 ± 6.19	0.73	-0.342	-0.217	2.9	7.15
Longest rally (s)	32.66 ± 10.97	37.00 ± 6.96	0.08	-1.739	0.425	2.98	5.32
Longest rally (strokes)	36.83 ± 8.38	41.25 ± 6.96	0.03	-2.086	0.546	4.39	5.93
Average rally (s)	7.58 ± 1.79	7.87 ± 1.20	0.84	-0.19	0.173	4.23	6.56
Average rally (strokes)	7.04 ± 1.11	7.75 ± 1.34	0.05	-1.928	0.61	6.34	5.78
Shuttles used	11.08 ± 4.63	12.00 ± 4.44	0.51	-0.655	0.2	2.39	2.7
**Set 1**							
Match points	*–*	*–*	*–*	*–*		*–*	*–*
Set points	1.65 ± 0.74	1.62 ± 1.40	0.65	-0.443	-0.032	2.23	1.16
Duration of set	18.58 ± 4.71	20.62 ± 4.12	0.33	-0.964	0.445	3.94	5
Total points played	35.79 ± 4.86	35.12 ± 3.24	0.32	-0.976	-0.147	7.36	10.84
Total point won	17.89 ± 4.60	17.56 ± 4.22	0.68	-0.409	-0.073	3.89	4.16
Most consecutive points	3.21 ± 1.67	2.75 ± 1.39	0.37	-0.888	-0.285	1.92	1.98
Longest rally (s)	29.62 ± 11.26	32.75 ± 7.47	0.90	-0.124	0.297	2.63	4.38
Longest rally (strokes)	32.54 ± 9.76	34.00 ± 10.23	0.84	-0.193	0.148	3.33	3.32
Average rally (s)	7.95 ± 2.11	7.87 ± 1.31	0.09	-1.698	-0.041	3.77	6.01
Average rally (strokes)	7.29 ± 1.28	7.87 ± 1.50	0.04	-2.057	0.436	5.7	5.25
Shuttles used	4.54 ± 1.91	6.12 ± 2.75	0.37	-0.884	0.744	2.38	2.23
Points scored without service	9.77 ± 2.29	10.56 ± 1.89	0.53	-0.621	0.358	4.27	5.59
Points scored with service	8.12 ± 3.94	7.00 ± 3.84	0.91	-0.109	-0.286	2.06	1.82
Biggest lead	5.51 ± 3.98	5.81 ± 3.94	0.16	-1.394	0.076	1.38	1.47
Biggest come back to win the game	2.20 ± 1.65	1.33 ± 0.57	0.63	-0.472	-0.587	1.33	2.33
**Set 2**							
Match points	1.75 ± 0.91	1.37 ± 0.74	0.71	-0.366	-0.434	1.92	1.85
Set points	2.00 ± 1.73	*–*	*–*	*–*		1.16	*–*
Duration of set	20.75 ± 4.53	22.00 ± 3.34	0.15	-1.429	0.291	4.58	6.59
Total points played	37.37 ± 5.24	34.50 ± 3.30	0.11	-1.562	-0.588	7.13	10.45
Total point won	18.68 ± 4.25	17.25 ± 4.52	0.26	-1.124	-0.332	4.4	3.82
Most consecutive points	3.84 ± 1.92	3.06 ± 1.56	0.47	-0.718	-0.422	2	1.96
Longest rally (s)	25.95 ± 7.52	32.75 ± 9.23	0.00	-2.799	0.858	3.45	3.55
Longest rally (strokes)	30.33 ± 7.32	38.00 ± 9.53	0.04	-2.004	0.976	4.14	3.99
Average rally (s)	7.70 ± 1.71	8.25 ± 1.61	0.50	-0.66	0.326	4.5	5.12
Average rally (strokes)	7.20 ± 1.23	7.87 ± 1.66	0.74	-0.328	0.501	5.85	4.74
Shuttles used	5.41 ± 2.08	5.87 ± 1.82	0.13	-1.489	0.227	2.6	3.23
Points scored without service	9.33 ± 2.56	9.25 ± 1.91	0.62	-0.495	-0.033	3.64	4.84
Points scored with service	9.35 ± 3.55	8.80 ± 4.41	0.86	-0.17	-0.146	2.63	2
Biggest lead	5.31 ± 3.57	6.33 ± 3.84	0.20	-1.266	0.281	1.49	1.65
Biggest come back to win the game	3.29 ± 2.02	3.00 ± 1.15	0.08	-1.748	-0.156	1.63	2.61
**Set 3**							
Match points	1.66 ± 0.51	*–*	*–*	*–*	*–*	3.25	*–*
Set points		*–*	*–*	*–*	*–*	*–*	*–*
Duration of set	24.50 ± 4.12	*–*	*–*	*–*	*–*	5.95	*–*
Total points played	33.35 ± 7.73	*–*	*–*	*–*	*–*	4.31	*–*
Total point won	10.08 ± 3.80	*–*	*–*	*–*	*–*	2.65	*–*
Most consecutive points	2.58 ± 1.16	*–*	*–*	*–*	*–*	2 .22	*–*
Longest rally (s)	30.16 ± 10.45	*–*	*–*	*–*	*–*	2.89	*–*
Longest rally (strokes)	32.33 ± 10.89	*–*	*–*	*–*	*–*	2.97	*–*
Average rally (s)	8.00 ± 1.59	*–*	*–*	*–*	*–*	5.03	*–*
Average rally (strokes)	7.33 ± 1.66	*–*	*–*	*–*	*–*	4.42	*–*
Shuttles used	4.50 ± 2.46	*–*	*–*	*–*	*–*	1.83	*–*
Points scored without service	10.83 ± 1.94	*–*	*–*	*–*	*–*	5.58	*–*
Points scored with service	7.25 ± 3.49	*–*	*–*	*–*	*–*	2.08	*–*
Biggest lead	5.37 ± 3.29	*–*	*–*	*–*	*–*	1.63	*–*
Biggest come back to win the game	2.00 ± 0.00	*–*	*–*	*–*	*–*	0	*–*


In the doubles modality, the results are more stable among groups. Men’s doubles had a longer duration of both match and Set (1, 2), as well as a longer average rally (Set 1, 3) and a higher number of shuttles used in the Eliminatory Phase vs. the Group Phase. Results for women’s doubles also show the number of shuttles used was higher in the matches in the Elimination than in the Group Phase. Moreover, the same results are established in Set 2, including for the average rally. Mixed doubles saw no match go to three sets. However, the greatest differences were found in the variables longest rally and average rally, which were higher in the Eliminatory Phase than in the Group Phase.

## Discussion

The main findings of this study show the big differences in the individual badminton between group stage vs. elimination phase, highlighting the differences in the third set. Doubles modality shows more stable results, standing out the non-existence of the third set in the mixed doubles.

In relation to the modality of singles, the match duration is longer in the EP vs. GP for both men (43–58 min) and women (40–50 min). Several authors have established a badminton match duration of between 40 and 50 min in men (Abian-Vicen et al., 2013; Gawin et al., 2015) and 17 and 28 min in women (Cabello-Manrique and González-Badillo, 2003; Cabello et al., 2004). This denotes a key important difference among players since one must be better prepared to face the eliminatory phase. In fact, Laffaye et al. (2015) found a duration of 78 min in men’s singles at the final of the London 2012 Olympics Games. Despite the importance of these results for player preparation, the data found in the set structure are more revealing. Results for men’s singles show differences between the GP and EP in Sets 1 and 3, with a duration from 18 to 29 min. Different authors have established the duration of a set at around 18 min (Abian-Vicen et al., 2013; Abián et al., 2014), hence making it important to see what happens between tournament phases, since the data reach almost 30 min in the EP. The same trend occurs in women’s singles, albeit as mentioned there are fewer values in the literature; in fact, data found for the Olympic Games are 12–13 min (Abian-Vicen et al., 2013), where higher values were recorded in Set 2. To our knowledge, this is the first study to show that Set 3 consumes the most time; with the figure for the EP even reaching 29 min, a fundamental consideration when planning training.

In addition to these data, some very interesting questions arise regarding the structure of shots. In men’s singles, in GP vs. EP, rallies have an approximate duration of 9–10 s, with 7.5–9 strokes per point, which occurs as a match average and even in Set 1 and Set 2. These findings are similar to those reported by other authors who indicated that high-level badminton entails similar data (Abian-Vicen et al., 2013; Abián et al., 2014). However, the differences between the GP and EP are decisive in Set 3 where the values reach 12 s in the EP, and 10.8 strokes per point. Thus, it is observed that the duration of a set gradually rises between the GP and EP and that the points tend to be longer with more strokes per point. In fact, in all cases, the stroke by time ratio has a tendency of 1 stroke every 1 second or every 1.12 s, which is one of the longest times so far various authors have determined a stroke frequency of between 0.56 and 1.08 (Alcock and Cable, 2009; Abian-Vicen et al., 2013; Gawin et al., 2015). It is interesting to note how more shuttlecocks are used in the EP, around 23 shuttles. But Set 3 in the EP stands out, reaching the highest values, with a value of 13 shuttles. Shuttlecock use relates to the force applied in execution of a stroke, mainly a smash, namely a common stroke in badminton (Abian-Vicen et al., 2013; Chiminazzo et al., 2018). This aspect was not evaluated in this study, but we showed that players entering the EP have at that stage already played longer sets, more strokes per point, and require more shuttlecocks. In addition, the longest rally is significantly higher in EP and more evident in Set 3 where it reaches a rally of 48 s with 47 strokes.

It is also interesting to note that the trend described in men’s singles is similar to women’s singles, except that the differences in average rally, duration, and strokes are greater in the EP, and increasing in all sets analyzed. Thus, the rally time is 9–10.50 s, with these figures exceeding those found in Olympic Games literature (Abian-Vicen et al., 2013; Abián et al., 2014) although similar for women’s world-class badminton (Gawin et al., 2015). Strokes per point hover around 6.6–7.5, consistent with earlier findings (Abian-Vicen et al., 2013; Abián et al., 2014). Yet, differences between phases are most evident in Set 3 where in the EP the point duration averages up to 13 s, with up to 10.50 strokes per point. It is even observed in Set 3 where the longest point is seen in the EP of up to 41 s, involving 36 strokes. This implies that in all cases, including the longest points, the ratio of strokes per time is around 1.1–1.2, below findings of other studies in girls showing 0.5–0.9 (Abian-Vicen et al., 2013; Abián et al., 2014; Gawin et al., 2015). These data are very revealing since, as indicated by several authors, the intensity of badminton involves playing around 90% of the time at the maximum HR (Álvarez et al., 2016; Bisschoff et al., 2016) so it is observed that it is not only necessary to reach the competition in good fitness but, as the championship progresses, the execution times and stroke volume tend to be higher, mainly in the EP and at key moments such as Set 3. Therefore, this indicates a very important change in men’s and women’s singles at the quantitative level between the GP and the EP, where Set 3 is key to sports performance.

As far as the game of doubles is concerned, the findings are useful in specialties attracting less scientific research. In men’s doubles, there is an increase in the match duration of 48 min in the GP compared to 68 min in the EP. Researchers have established an average of 40–45 min (Gawin et al., 2015), although longer lasting matches have been observed over the last three Olympics (Abián-Vicén et al., 2018). However, the fact the matches are longer in duration in the EP implies the need to better prepare for the final part of the tournament. To our knowledge, no specific data are available about the duration of a set, where in the present study some interesting questions arise. On one hand, the duration is significantly longer in Set 1 (18 vs. 23 min) and Set 2 (20 vs. 29 min) between the GP vs. EP. Albeit not significantly, statistically speaking, Set 3 has a longer duration in the GP vs. EP (25 vs. 22). Therefore, this seems to show a set duration of around 20 min, similar to a duration calculated based on others’ findings (Gawin et al. (2015) and, over and above the data that exists in singles match (Abian-Vicen et al., 2013; Abián et al., 2014). The level of EP is similar, but in high-level men’s doubles, it seems there were greater differences in superiority in the modality of partners and hence the Set time is lower.

The duration of a point, despite a tendency to rise between the GP vs. EP, is not significant in either a match or any analyzed set. Thus, the duration of a point is between 6 and 7 s, with 7–7.6 strokes per point. The duration of a point has grown over time, from 5 s established some time ago (Liddle and O’Donoghue, 1998; Alcock and Cable, 2009), until the interval established by other researchers (Gawin et al., 2015; Abián-Vicén et al., 2018). With regard to strokes per point, different authors have determined a range of 8–10 (Abián-Vicén et al., 2018). The highest values are found in Set 2, in the EP, with 8.58 strokes per point. This seems to be high since the first three strokes are decisive in men’s doubles, where 80% of attack maneuvers begin with the service-return stroke (Gawin et al., 2013). These data give an approximate stroke-to-time ratio of 0.85–0.90, slightly higher than that found on a world-class level (Gawin et al., 2013). At the last three Olympics, a stable trend of around 1.5 is apparent (Abián-Vicén et al., 2018), which appears to be a high ratio given that the network game, is higher in doubles games is more evident (Zhang et al., 2013). Observing the structure by Set and by phase of competition may produce some results that can underpin modality-specific training.

There are almost no differences between the GP and EP in women’s doubles. The longer match duration in the EP vs. the GP (68 vs. 47 min) stands out. Of the few studies that concern women’s doubles, Gawin et al. (2015) found a duration of around 40 min. At the Beijing and London Olympic Games, durations of 42–47 min were observed similar to 47 min in the GP in this study, but away from the time in the EP. Abián-Vicén et al. (2018) note that, after the Hawk-eye appeared in badminton, observed rest times have increased, which may explain this increase when the other variables, as we will see below, do not show significant differences. An increase in the Set duration in the GP vs. the EP is shown, but it is not significant, placing it around 20–24 min, longer than what happens in women’s singles (Abian-Vicen et al., 2013). Thus, in women’s doubles there are no differences in the GP and EP, in the point duration, in strokes per point, in the longest points, or in the match duration in each of the sets analyzed. The average point is around 10–11.50 s with an average of 10–11.50 strokes per point, indicating an approximate ratio of 1:0. The duration of a point in women’s doubles is established at around 7–10 s (Gawin et al., 2015; Abián-Vicén et al., 2018), showing progress made in the sport which a decade ago and, prior to the regulatory change, for this had values around 5 s (Liddle and O’Donoghue, 1998; Alcock and Cable, 2009). Strokes per point appears to have a value of approximately 12 (Abián-Vicén et al., 2018), slightly higher than our results, although it is true they are global data and the fact the present study was performed by competition phases and Set may explain the difference. If it is true, a stroke time ratio with a stable value of 1:2 is established (Alcock and Cable, 2009; Abián-Vicén et al., 2018), also above what is determined here. It calls for attention because, like in men’s doubles, in women’s, the first three strokes are vital, with 50% of attack actions being initiated after the return of service (Gawin et al., 2013). While some might say the level of experience in men players is very evident, this is not so much the case for women players (Gawin et al., 2013).

In badminton, doubles players are specific in that they do not participate in singles competitions as may happen in other sports like tennis (Torres Luque and Carrasco Páez, 2004). Along these lines, no great differences are observed between tournament phases, possibly indicating the level of competition is similar between partners in high-level competition, an aspect that deserves to be analyzed in more depth.

In mixed doubles, what is most striking is that at the Rio Olympic Games no match was played to three sets, revealing the constant superiority of certain partners compared to others. In fact, unlike in men’s and women’s doubles, mixed doubles show a difference between the GP vs. the EP. Particularly standing out is strokes per point at around 7.8, with the duration of a point impacting those values. The duration of a point in strokes at this level is already higher than about 5 and 5.6 found by other authors at lower levels of competition (Liddle and O’Donoghue, 1998; Gawin et al., 2015), although the stroke per point ratio extracted in this study exceeds 0.72 determined by Gawin et al. (2015). Considering the scarcity of scientific studies on this sport, it is necessary to regard these data as a reference for what is currently happening at high levels of the sport and to continue to investigate in the future.

A strength of this study is the analysis of competition statistics for five badminton modalities, where differences are observed in levels of competition and analyzed by set.

The applications of this study to specific training in a badminton sport, could be defined in two fundamentals: on the one hand, the differences in group phase vs. elimination phase, indicated that in high performance there are two competitive levels. This aspect, which is observed more in the singles badminton, so that, when planning competitive periods of high level, this must be considered. On the other hand, and we consider that more novel, is the analysis of these two phases and by set. So far, we only had analysis of set 1 and set 2, but few studies on set 3. This implies observing as set 3, which is determinant for the winner the match and therefore, to obtain medal in a competition such as JJOO, is substantially different between group stage vs. elimination phase. This study can contribute to a more specific preparation about being good or being the best.

## Conclusion

The results show in men’s and women’s singles all the variables related to the match were higher in the Eliminatory Phase than in the Group Phase. Sets 1 and 3 registered the longest set duration, rally and average rally in the Eliminatory Phase. In women’s singles, these differences were also established in set 2. In doubles, the results are more stable among groups. Men’s doubles had a longer match duration and set (1, 2) duration, and scored higher for average rally (sets 1, 3) and shuttles used in the Eliminatory Phase vs. the Group Phase. In women’s doubles, more shuttles were used in a match in the Eliminatory Phase than in the Group Phase. Moreover, the same results are found in set 2, including the average rally. Mixed doubles did not see any match go to three sets. However, the biggest differences were found in the variables longest rally and average rally which were higher in the Eliminatory than in the Group Phase. This information may help players and coaches prepare and administer different types of workouts or, more specifically, competition schedules adapted to the characteristics of modern badminton.

## Author Contributions

GT-L and MK conceived and designed the study. GT-L and ÁF-G analyzed the data. JB-T and GT-L drafted the manuscript. DC-M and MK advised on analysis and interpretation of the data and critically revised the manuscript. DC-M funding acquisition.

## Conflict of Interest Statement

The authors declare that the research was conducted in the absence of any commercial or financial relationships that could be construed as a potential conflict of interest.

## Footnotes

1 https://www.olympic.org/badminton
